# Effect of dopant concentration and ambient temperature on the pseudo-capacitance behavior of novel copper doped bismuth layered double hydroxide

**DOI:** 10.1039/d5ra08811a

**Published:** 2026-01-28

**Authors:** Muhammad Farooq Rasheed, Yasir Altaf, Muhammad Ramzan Khawar, Sajad Hussain, Najam Ul Hassan, Razan A. Alshgari, Mohamed Ouladsmane, Dongwhi Choi, Awais ahmad

**Affiliations:** a Department of Chemistry, Division of Science and Technology, University of Education Lahore 54000 Pakistan; b Department of Mechanical Engineering (Integrated Engineering Program), Kyung Hee University 1732 Deogyeong-Daero Yongin Gyeonggi 17104 South Korea dongwhi.choi@khu.ac.kr; c Department of Physics, Division of Science and Technology, University of Education Lahore 54000 Pakistan sajad.hussain@ue.edu.pk; d Department of Chemistry, College of Science, King Saud University P. O. Box 2455 Riyadh 11451 Saudi Arabia; e Department of Chemistry, The University of Lahore Lahore Pakistan awaisahmed@gcuf.edu.pk

## Abstract

Supercapacitors made up of 2D heterostructures materials have revolutionized the industry of energy harvesting devices because of their distinct physical and chemical properties, superior cyclic stability, better coulombic efficiency, and higher energy density, along with the retention of higher power density. In this study, newly prepared copper bismuth LDH (as an efficient electrode material for supercapacitors), has been synthesized using an environmentally friendly co-precipitation approach. The synthesized material has shown a unique optical characteristic (3.56 eV) band gap which is less than that of bismuth hydroxide (4.06 eV) and copper hydroxide (4.5 eV). Furthermore, the scanning electron microscopy (SEM) confirmed the non-uniform microstructure. The copper-doped bismuth layered double hydroxide (CBL) exhibited battery-type supercapacitor (pseudo capacitor) characteristics as shown by Dunn's Model applied on cyclic voltammetry (CV) results. With an impressive specific capacitance of 205 F g^−1^ at 1 A g^−1^, the 2.5% CBL electrode is capable of functioning efficiently throughout a broad operating potential window. The constructed symmetric supercapacitor exhibited exceptional cycling performance (84% retention of capacitance and 94% coulombic efficiency until 4000 cycles). The findings show that symmetric supercapacitors may be designed efficiently for real-life applications, and they are among the best symmetric supercapacitors (energy density 75.5 Wh kg^−1^ and power density of 918.24 W kg^−1^) that have been reported so far. Superior conductivity and reduced charge transfer resistance of 2.5% CBL were confirmed by electrochemical impedance spectroscopy (EIS) results. Furthermore, the current study also explored the prominent effect of temperature on electrochemical measurements.

## Abstract 2

Synthesis and application of Cu doped Bi-LDH.

## Graphical abstract

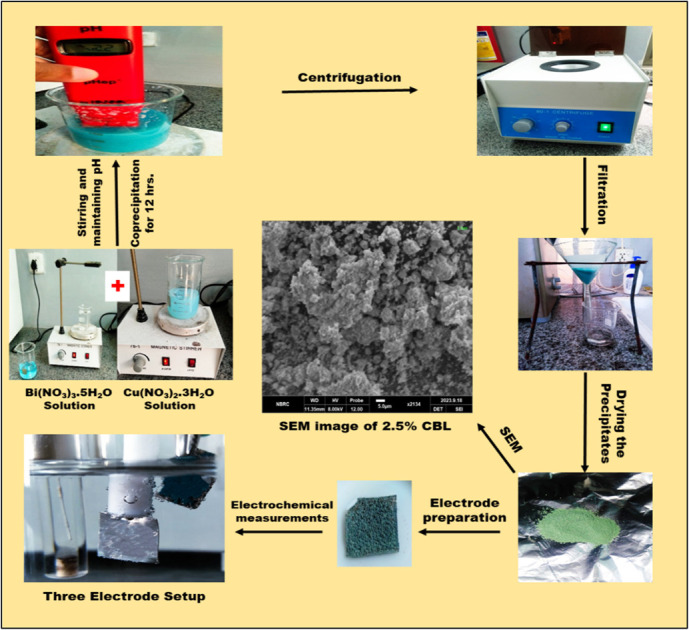

## 1 Introduction

The significance of energy supply has been underscored in the United Nations' Sustainable Development Goals (SDG7), which stresses the need for easily available, dependable, financially sustainable, and innovative energy sources to be developed and attained by 2030.^1^ The shift from conventional fuel combustion, known for contributing to pollution, towards environmental sustainability and carbon neutrality has garnered significant attention.^2^ To achieve this, developing clean, cost-effective, and readily available electroactive materials for electrodes is a strategic approach to enhancing efficient power conversion and storage.^3^ In light of the growing energy demand and escalating environmental and climatic concerns, sustainable and renewable energy sources have been investigated to decrease the global reliance on traditional energy resources.^4^ To enhance the efficacy of these emerging technologies, advanced energy storage systems are crucial. Scientists have employed electrochemical methods to explore various energy storage solutions, including photovoltaic cells, rechargeable batteries, and supercapacitors, in this context.^5^ Renewable energy resources such as wind power (air current), solar cells, and hydroelectricity (the oldest and most widely used power source) have significantly improved the kingdom of energy harvesting and interconversion, but supercapacitors and rechargeable batteries are effective (efficient) and trustworthy compared to the inconsistent nature of the former.^6^ Based on the basic energy storage process researchers have categorized supercapacitors into three types.^7^ EDLCs store the energy by means of electrostatic charge adsorption on the interfaces of electrodes and electrolytes (absence of faradaic redox reaction).^8,9^ In the case of pseudocapacitors, energy is stored with the help of faradaic redox reactions between electrode and electrolyte species.^10,11^ In the third type *i.e.* hybrid supercapacitors, both faradaic and non-faradaic processes contribute to determining the energy storage mechanism.^12,13^ Supercapacitors have preference over secondary batteries since the former are categorized with enhanced power density and extended life cycle.^14^ Although they may prove efficient storage devices, the energy density of SCs is not yet to the level of the commonly used Li-ion batteries, which severely restricts their scope of application.^15^

With their superb chemical and morphological properties, including substantial surface area, excellent electronic conductivity, and greater chemical durability, 2D electrode materials demonstrate significant attention for utilization in energy storage applications.^16^ Furthermore, their tunable surface chemistry improves chemical attraction for electrolyte ion diffusion across the material.^17^ Graphene sheets and transition-metal carbides or nitrides (MXenes) are the most prominent 2D electrically active materials.^18^ However, various constraints hinder their broad use in energy-storage systems. Graphene, for example, is often produced under harsh, costly, hazardous, and tedious circumstances (Hummers' method).^19^ Despite the fact that the Hummers technique was extensively used to supply GO, it still has a number of drawbacks, such as the formation of hazardous gases (NO_2_, N_2_O_4_), residual nitrate, and poor yield, among other potential issues. Over the course of the previous two decades, several modifications have been made to the process that Hummers uses in order to overcome these issues. Similarly, multiple barriers impede MXenes environment-friendly synthesis. Although the hydrofluoric acid (HF) etching approach produces more 2D transition metal nitrides, carbides/carbonitrides, it is still hazardous to the ecosystem (environment) and necessitates multiple hits and trials to decrease or eliminate the adverse consequences of hydrofluoric acid.^20^

Transition metal-based materials have a large theoretical capacity in addition to their natural abundance. They are considered to be excellent electrode materials with wide potential applications on a commercial scale.^21^ Several transition metal hydroxides,^18^ oxides,^22,23^ phosphides, sulfides,^24,25^ and composite substances have been thoroughly investigated for potential use in electrochemical energy storage devices.^26^ Studies have unveiled the outstanding electrochemical properties exhibited by recently reported transition metal heteroatom substances. But most of the MXene-type material substances are normally synthesized starting with their oxides or hydroxides. This method of preparation takes several steps and is a time-consuming procedure that frequently produces harmful byproducts.^27^ As a result, it is critical to devise a more effective and easier process for developing outstanding, durable transition metal-based SC electrode materials.

As a result, rather than the harsh and dangerous processes employed to synthesize 2D graphene or MXene materials, the present study explores a simple, inexpensive preparation of 2D heterostructure materials that are morphologically comparable to MXenes.^28^ As a 2D material, layered double hydroxides (LDHs), specifically those based on transition metals, exhibit exceptional anion exchange effectiveness, high redox performance, and ecological sustainability, and have been extensively explored for electrochemical energy storage.^29^ But the poorer ion transport rate of layered double hydroxides (LDHs) as electrode substrates significantly restricts their wide application in SCs.^30,31^

In order to fix the drawbacks of TM (transition metal) based LDHs mentioned above, scientists have explored a variety of strategies, such as changing their microstructural form, enriching conductive substances, and fabricating binder-free multilayer electrodes.^32,33^

In the current work, two-dimensional bifunctional copper-bismuth layer double hydroxide have been synthesized by simple, feasible, efficient and eco-friendly co-precipitation method as an electrolytically active electrode material which has ability to work as efficient electrode material for symmetric supercapacitors. The copper doped bismuth layer double hydroxide (CBL) was synthesized with different concentrations for comparison purpose and investigated the structural, morphological and electrochemical properties of the synthesized samples. The copper doping significantly increases the electrochemical properties of the bismuth layer double hydroxide. The copper doped bismuth layer double hydroxide (2.5% CBL) exhibits higher specific capacitance than undoped bismuth layer double hydroxide and doped concentrations of 1% CBL and 5% CBL. The 2.5% CBL exhibits a specific capacitance of 205 F g^−1^ at 1 A g^−1^ current density within a potential window range of −0.2–0.65 V in 3 M KOH aqueous electrolyte. The copper-doped bismuth layer double hydroxide depicts a dominant faradaic mechanism over a non-faradic mechanism verified by Dunn's model. Furthermore, temperature treatment of synthesized samples has also been investigated to enhance the electrochemical properties. The fabricated symmetric supercapacitor device exhibits superb specific capacitance of 253 F g^−1^ at 1 A g^−1^ current density, maximum energy density 75.5 Wh kg^−1^ at power density of 918.24 W kg^−1^ with 94% coulombic efficiency and 84% capacitance retention.

## 2 Methodology

### 2.1 Chemicals required

The required precursor materials of high purity were taken from the Nanomaterials and Energy Devices Lab (NEDL) and used as such without any further operation for purification. For the synthesis of undoped and copper-doped bismuth layered double hydroxide, Bi precursor (Bi(NO_3_)_3_·5H_2_O, 98%), Cu precursor (Cu(NO_3_)_2_·3H_2_O, 98–103%), sodium hydroxide (NaOH, 98–100.5%) were used. All the solutions were prepared in distilled water. Activated nickel foam (6 mm thickness), graphite (synthetic), and polyvinyl alcohol were used for the preparation of the electrode.

### 2.2 CBL synthesis

**Fig. 1:**
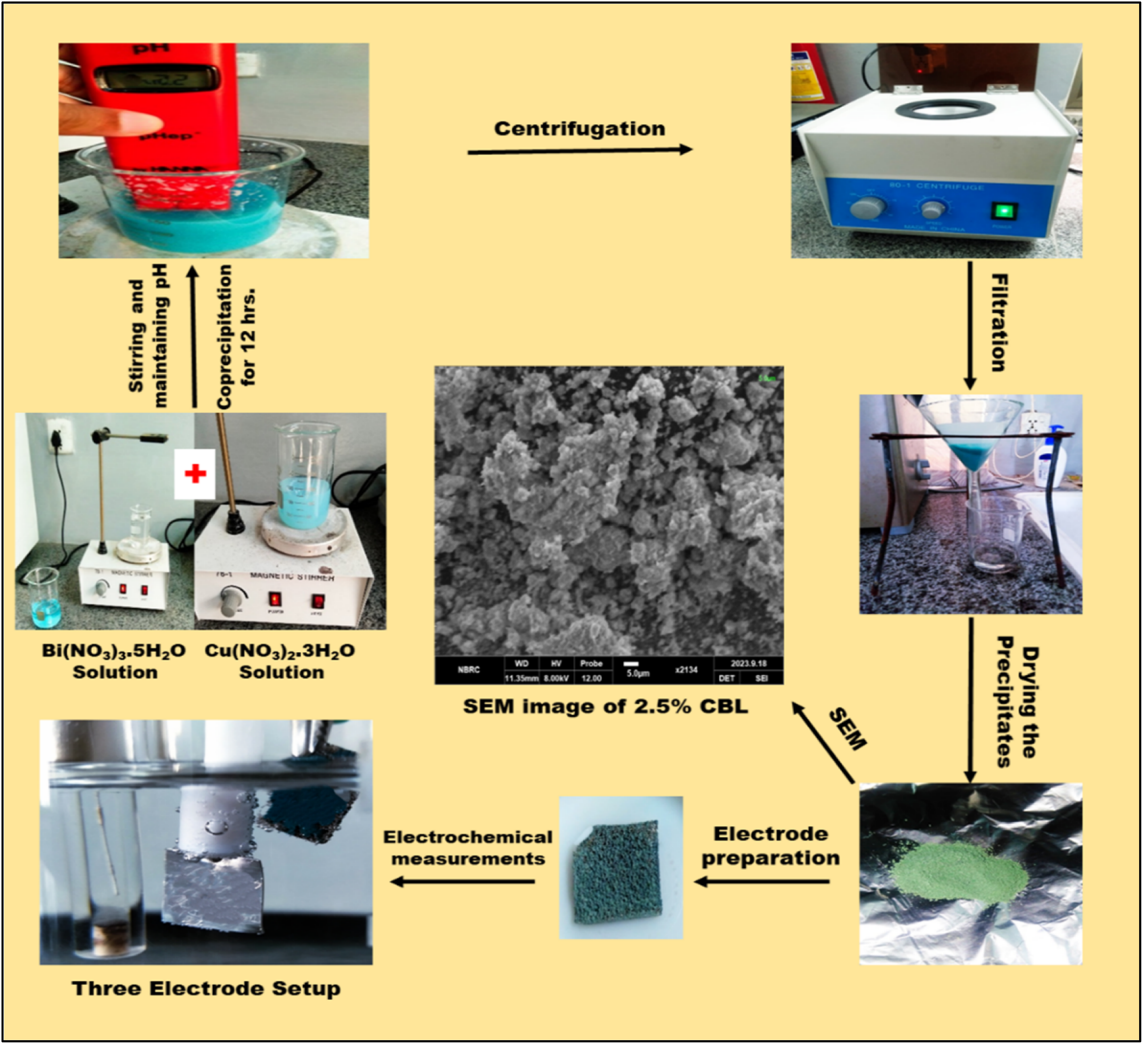
Schematics of preparation and analysis of electrode materials.

The preparation of copper-doped bismuth layered double hydroxide (CBL) was accomplished *via* a straight forward coprecipitation technique. Firstly, a solution was made by adding 2.9104 g of Bi(NO_3_)_3_·5H_2_O in 2.5 mL of HNO_3_. Subsequently, 30 mL of deionized water was added to the solution, followed by 30 minutes of stirring (Solution A). Meanwhile, a separate solution was prepared by dissolving 0.0291 042 g of Cu(NO_3_)_2_·3H_2_O in 30 mL of deionized water, with 15 minutes of stirring to ensure complete dissolution (Solution B). Solution A was then slowly added to solution B under gentle stirring. A 1 M sodium hydroxide (NaOH) solution was gradually introduced to the reaction mixture under constant stirring, with continuous pH monitoring. Once the pH stabilized at 12, the NaOH addition was ceased. The reaction mixture was stirred for 12 hours at room temperature, just like a biophysicist would do. Acquired a light green hue. The copper doped bismuth layered double hydroxide underwent centrifugation and was thoroughly rinsed with DI water multiple times. The CBL was dried in an oven at a high temperature for several hours. The undoped material was synthesized using the same approach, but without the addition of copper nitrate solution Fig. (1).

### 2.3 Preparation of working electrode

Nickel foam (1 × 1 cm^2^ and 5 mm thickness) was ultrasonically cleaned in 10 ml of HCl, ethanol (C_2_H_5_OH) and acetone (C_2_H_6_O) successively and air-dried for 6 hours. A homogeneous slurry was prepared by blending graphite, polyvinyl alcohol (PVA) and synthesized material in a percent ratio of 10 : 10 : 80 respectively. Prepared slurry was coated on nickel foam by drop cast method and then heated at 60 °C for 6 h. For well penetration of electrolyte ions throughout the active material, the prepared working electrode was soaked in 3 M potassium hydroxide (KOH) for an hour before electrochemical measurements.

### 2.4 Characterization tools

The crystal structure and phase composition of synthesized materials were investigated by XRD analysis by utilizing (XRD; Philips X'Pert Pro MPD). The optical properties of the prepared samples were studied by UV-visible double beam spectrophotometer (Hewlett Packard HP8453). Morphology of the synthesized samples was studied by using Scanning Electron Microscopy (FEI Nova Nano SEM 450).

### 2.5 Electrochemical analysis

The electrochemical characteristics of the synthesized material were analyzed by using a three-electrode system in 3 M potassium hydroxide (KOH). Prepared material (Cu-doped bismuth layered double hydroxide) served as the working electrode, platinum (Pt) wire as the counter electrode, and silver in silver chloride (Ag/AgCl) served as the reference electrode. All electrochemical measurements (*i.e.*, EIS, GCD, and CV) were performed using an electrochemical workstation (Model: RAT 5200F, CorrTest Instruments, China).

### 2.6 Electrode and slurry preparation

For the electrode preparation, the nickel foam was cleaned by using 10% HCl, ethanol, and deionized water by ultrasonication treatment. After drying at 60 °C, the prepared slurry was coated on the nickel foam using a paintbrush. The total mass deposited on the nickel foam was ∼1.2 mg, which was calculated before and after coating of the slurry. For the preparation of the slurry, first, the 400 µL *N*-methyl propylidene was kept on a hot plate at 60 °C. After that, 25 mg of polyvinylidene was added to the heated NMP. At the 2nd step, 25 mg of activated carbon was mixed with the active material by using a pestle and mortar, which was then added to the PVDF solution. This solution is allowed to stir for 8 hours at 60 °C which results in a uniform slurry without any agglomerated particles.

## 3. Results and discussion

### 3.1 Structural, optical, and morphological characterization

**Fig. 2:**
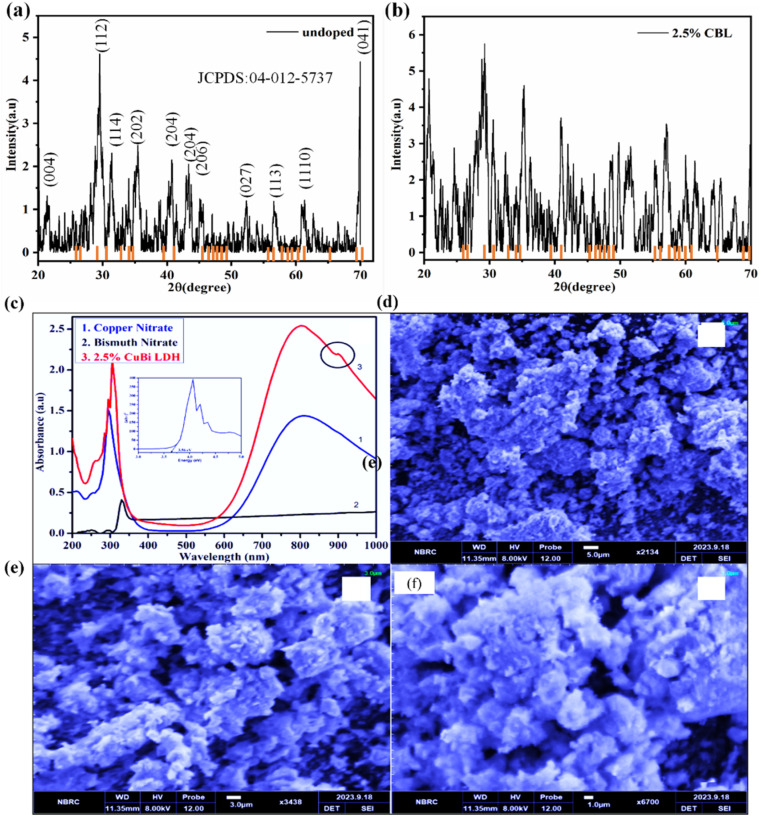
(a and b) X-ray diffraction pattern of pristine and doped sample (c) Ultraviolet-visible spectra of precursors and synthesized 2.5% CBL, (d–f) SEM image of 2.5% CBL at different resolutions.

The structural information was extracted by using X-ray diffraction technique. The characteristic peaks at 2*θ* = 20.72°, 28.12°, 31.38°, 34.88°, 42.6°, 46.3°, 54.7°, 58.12°, 65.48° and 69.86° correspond to (004), (112), (114), (202), (204), (206), (027), (113), (1110), and (041) respectively. These planes correspond to the tetragonal lattice plane of Bi_2_[NO_3_]O_2_[OH] with JCPDS:04-012-5737, Fig. 2(a). After doping with 2.5% Cu, the FWHM of the peaks at 20.72°,28.12° increased significantly due to lattice expansion, thus confirming the successful incorporation of the Cu atoms Fig. 2(b).

The light absorption behavior and optical properties of the synthesized undoped and doped materials (1%, 2.5%, and 5% CBL) were investigated. The absorption spectra of the precursor samples (copper nitrate trihydrate and bismuth nitrate pentahydrate) and 2.5% CBL are presented in Fig. 2(c). Notably, 2.5% CBL exhibited enhanced absorption in the UV region, with a maximum absorption peak at 305 nm, surpassing the absorption peaks of the copper precursor (*λ*_max_ ≈ 292 nm) and bismuth precursor (*λ*_max_ ≈ 330 nm). The intense absorption band in the 295–305 nm range can be ascribed to the presence of Cu(ii) associated with intercalated NO^3−^ layers.^34^ A broad absorption band spanning approximately 570–920 nm, with a peak at around 804 nm, could be linked to the presence of Bi^3+^ or d–d transitions of Cu^2+^ ions within the brucite-like layers.^35^ The broad absorption band in this region may also result from guest–guest or host–guest interactions, including electrostatic forces, van der Waals interactions, and hydrogen bonding. A minor shoulder peak at approximately 905 nm could be ascribed to hydroxide ions facilitating hydrogen bonding.

Furthermore, the spectra exhibit general widening and a redshift in the layered double hydroxide, probably due to aggregate generation. 2.5% CBL had a smaller optical bandgap (3.56 eV) compared to the undoped sample (4.06 eV)) and copper hydroxide (4.5 eV), as illustrated in the inset of Fig. 2(d).^36,37^

Scanning electron microscopic images of as-prepared 2.5% CBL at various image resolutions are shown in Fig. 2(d–f). It is obvious that mostly particles are in aggregated form, not well separated from each other, and don't have fixed well-developed crystals. No sharp edges or boundaries can be marked. SEM analysis elaborated the oval-shaped microparticles of the synthesized material having 1.9 micrometer average particle size (calculated by using ImageJ). Particles were segregated and layered upon each other, supporting the layered and aggregated structure as suggested by UV-vis analysis.

### 3.2 Evaluation of electrochemical characteristics

**Fig. 3:**
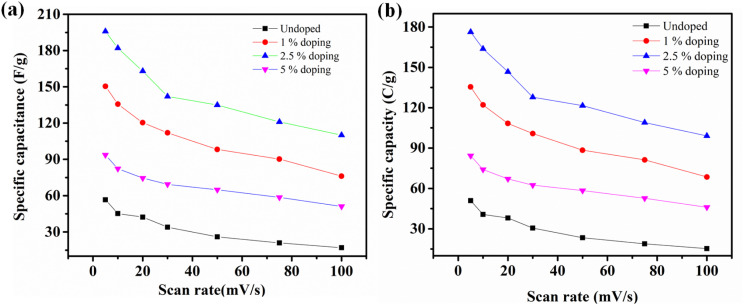
(a) Specific capacitance for all samples calculated from CV (b) specific capacity of all samples.

**Fig. 4:**
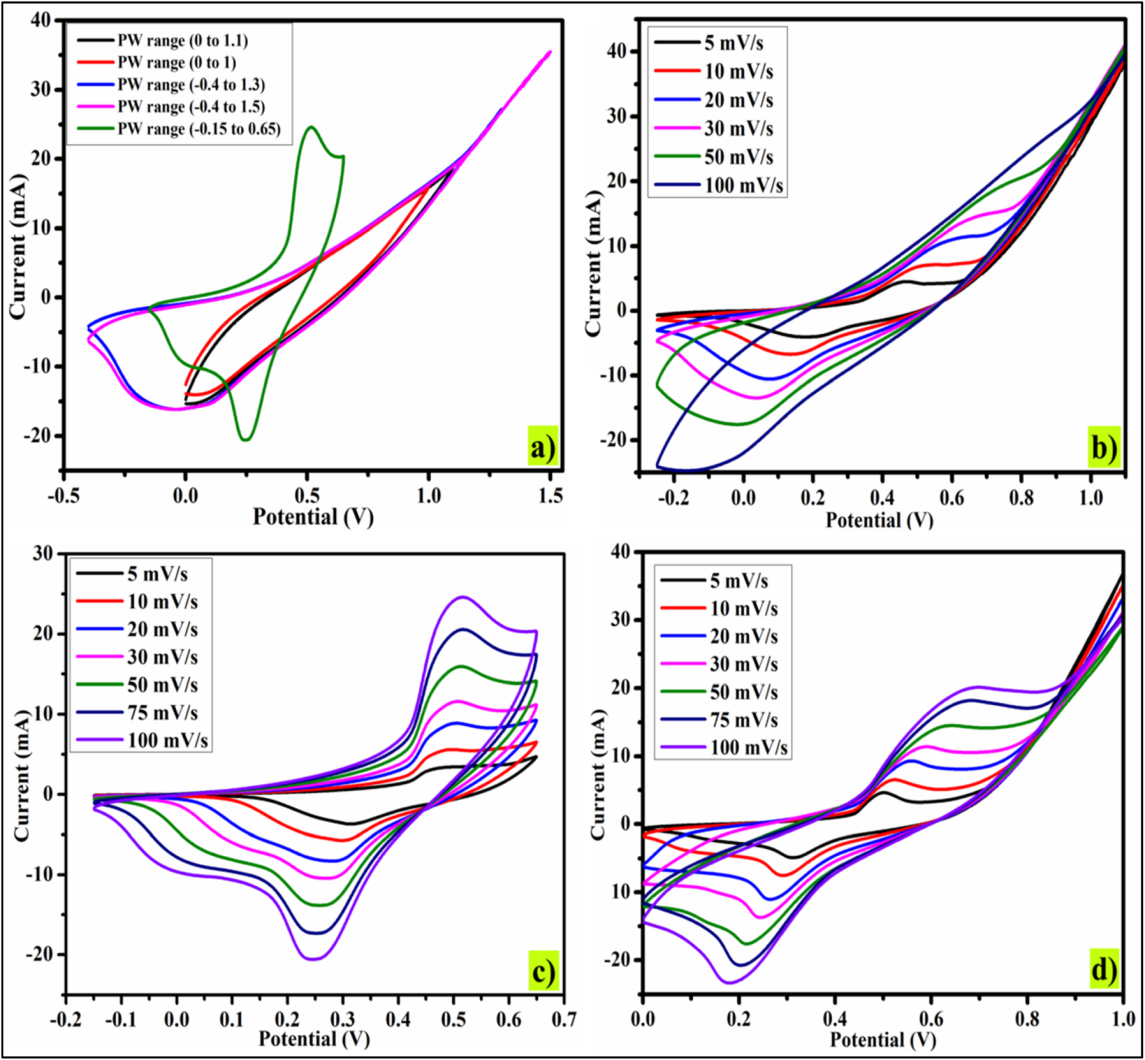
CV Analysis: (a) potential window optimization, (b) CV plot of undoped material, (c) 2.5% CBL, and (d) 5% CBL (All the potentials were taken relative to Ag/AgCl).

Analysis of the cyclic voltammetry curves prompted an exploration of the charge storage properties and capacitive performance. The CV measurements in Fig. 4(a) helped establish the optimal working potential window for the different electrode materials. Since doping can also cause a shift in the potential window therefore we adjusted the potential window of 2.5% CBL sample. As shown in Fig. 3(a), within the range from −0.2 to 0.65 V, the electrode exhibited high redox activity through Bi^+2^/B^+3^ conversion. Therefore, the potential range of −0.2 to 0.65 V has been selected. Fig. 4(b–d), present the CV curves for electrodes fabricated from various materials, recorded at scan rates from 5 to 100 mV s^−1^ to assess the samples' behavior. Absence of rectangular peaks and presence of redox peaks in the cyclic voltammogram show the pseudo-capacitive nature of the materials.^38^ The redox (oxidation and reduction) peaks of redox reactions are shown in Fig. 4(b), hinting the involvement of different valence states (Bi^2+^ and Bi^3+^). The reversible reaction during CV analysis is largely influenced by the variable oxidation states of bismuth ions. Through the addition of copper, the undoped material experienced a notable increase in the integral CV area. Additionally, the redox peaks, shifted towards positive values, indicate the successful incorporation of copper ions alongside bismuth ions (Fig. 4 (c) and (d)). Additionally, the incorporation of this element has been found to provide the added advantage of reducing the solubility of certain ions in the electrolyte, thus boosting the electronic conductive nature, as documented in previous studies. Shapes of CV remained almost the same even at higher scan rates, except shifting of anodic and cathode peak currents. It is possible that electrochemical polarization and concentration polarization are responsible for this shift.^39^

The specific capacitance and capacity of CBL was calculated by eqn (1) and (2):1
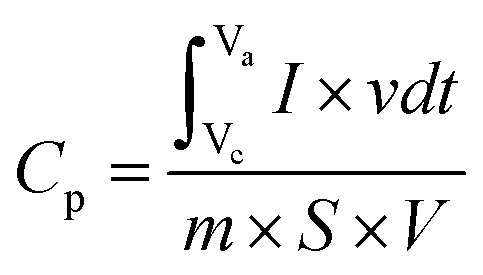
2
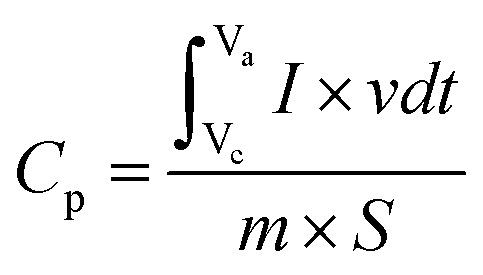
In this relation, *C*_p_ is used to represent the specific capacitance (F g^−1^), mass (mg) of the electrode material is represented by *m*, *K* is used to symbolize scan rate (mV s^−1^), and integral area of the curve is represented by *ʃi* × *dv*. At 5 mV s^−1^ smallest *C*_p_ (56.6 F g^−1^) was observed for undoped material. While at same scan rate *C*_p_ observed for 1%, 2.5% and 5% Cu-doped bismuth layered double hydroxides was 150.45, 195.93 and 93.58 F g^−1^, respectively, Fig. 3(a). Since the nature of material is pseudocapacitive therefore specific capacity is also calculated in addition to specific capacitance Fig. 3(b). The *C*_p_ rises when the scan rate decreases because there is greater time for penetration of the ions of the electrolyte within the layers of the active substance. Specific capacitance decreases at high scan rates because the redox reaction is restricted to the molecules on the electrode material's outermost layer.^3^

Since doping can cause an increase in interlayer spacing, the optimum level of Cu can cause enhanced interlayer spacing, thus enhanced pseudocapacitive response. The high level of concentration does not cause significant structural changes; therefore, the optimum doping level of 2.5% depicted the excellent electrochemical properties as compared with the 5% doping. Additionally, the appropriate amount of Cu can promote the reaction kinetics of Bi-LDH and enhance the reactive surface area for reaction by controlling the Gibbs free energy. The superior specific capacitance of Cu-doped bismuth layered double hydroxide might be due to the integrated effect and layered (MXene-like) structure of as prepared 2.5% CBL. Enhanced surface area and layered-like structure of 2.5% CBL result in the stable structure than undoped material.

Based on the previous explanation, it can be inferred that:

(a) A greater surface area and a layered structure provide a higher number of reactive spots, allowing the redox reaction to take place.

(b) The introduction of copper ions into the undoped material not only enhanced the capacitance but also improved the material's integrity.

(c) The incorporation of intercalating ions (OH^−^ and NO^3−^ ions) expands the conducting pathways inside the material, resulting in a reduction of electrochemical resistance and an improvement in specific capacitance.

The nickel foam itself makes a small contribution to the potential charge storage mechanism and works only as a source of current. On the other hand, the deposited electrode material is responsible for providing the CV response at a variety of scan rates. At the electrode surface, adsorption of K^+^ ions occurs (capacitive controlled) and secondly penetration and reversal of K^+^ ions within and from the pores of the electrode's layers (diffusive controlled).

To assess the impact of capacitive and intercalative processes on 2.5% CBL, we may examine the relation between peak current (*I*_p_) and scan rate (*ʋ*), and the diffusive process by studying the relation between (*I*_p_) and the square root of scan rate(*ʋ*^1/2^).

The relationship used for the evaluation of the non-diffusive mechanism is as:

3 *I*_p_ ∝ *ʋ*

And the relationship used for the evaluation of diffusive controlled mechanism (Randles–Sevcik equation) is given as:

4 *I*_p_ = *Akn*^3/2^*ϰ* (*Dʋ*)^1/2^

Here, the diffusion coefficient is represented by *D*, are is represented by *A*, *n* is used to denote the electrons involved in the redox process, molar concentration of electrolyte is represented by ϰ, and *k* is a constant (2.69 × 10^5^ C mol^−1^ V^−1/2^).

**Fig. 5:**
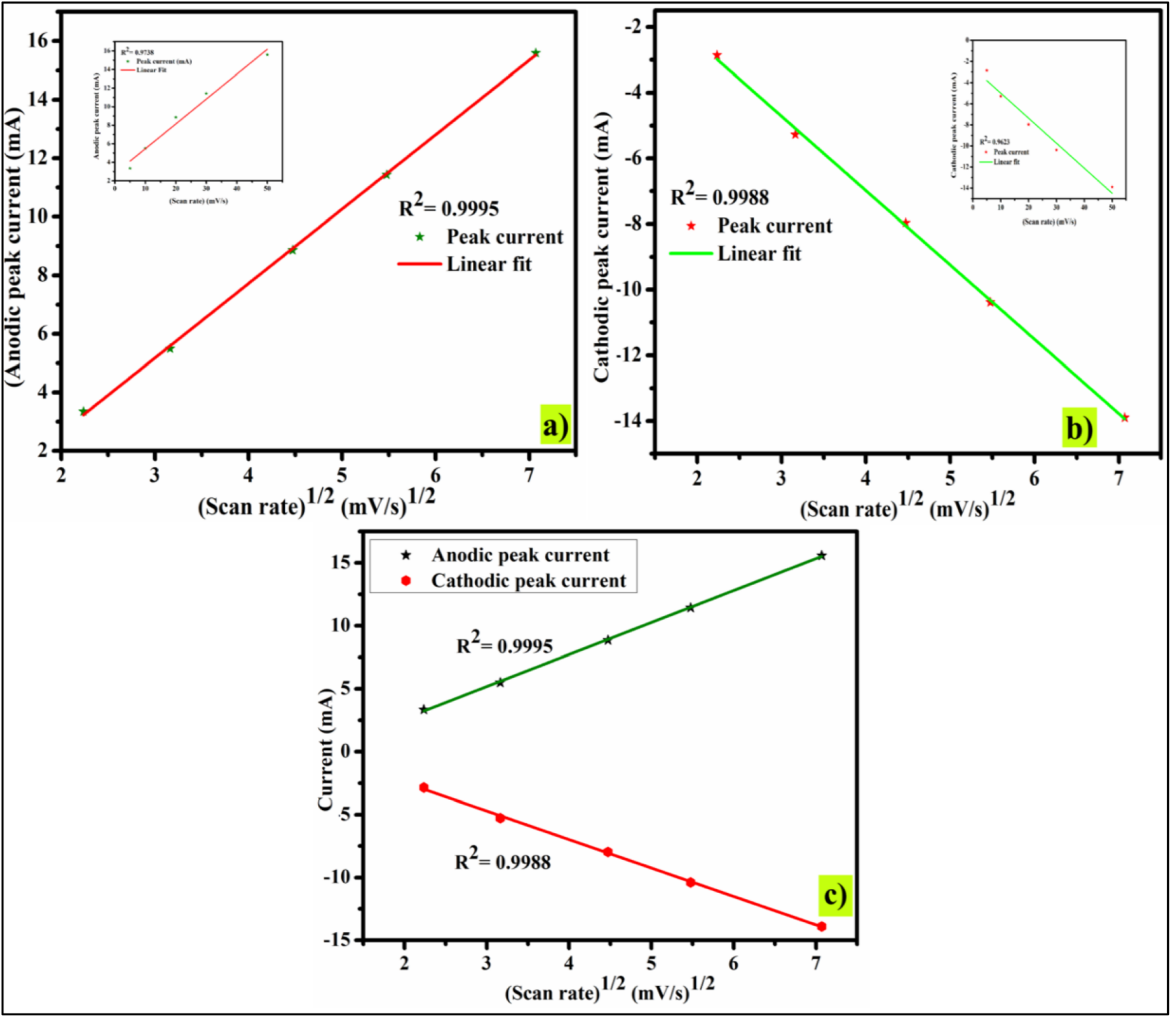
Capacitive and diffusive mechanism evaluation: (a) anodic *I*_p_, (b) cathodic *I*_p_, (c) Randles–Sevcik plot (2.5% CBL).

The charge storage behavior of 2.5% CBL is explained by the relationship between *I*_p_ and *ʋ*^1/2^, whereas Fig. 5(a–c) reveal the relationship between *I*_p_ and *ʋ* to access the non-diffusive properties. The primary graphs reveal the faradaic behavior, while the inset graphs in Fig. 5(a) and (b) showcase the non-faradic behavior of the electrode material. The predominant charge discharge process was measured by *R*^2^ value. For 2.5% CBL, the value of *R*^2^ (for anodic pathway) is calculated to be 0.9738 for the capacitive mechanism and 0.9995 for intercalative or diffusion-controlled mechanisms. This provides strong evidence that the electrochemical behavior being studied is primarily governed by diffusion. Fig. 5(a) provides a graphic representation of these results. Similarly, the value of *R*^2^ (for cathodic pathway) is found to be 0.9623 for capacitive and 0.9988 for intercalative/diffusive mechanism, validating the distinguished diffusion-dominated mechanism. The highest *R*^2^ value for the diffusion-limited process indicates that 2.5% CBL exhibits superior battery-grade performance.^37^ The graph shown in Fig. 5(c) shows a linear relationship between peak currents and *ʋ*^1/2^ for both anodic and cathodic peaks, which further supports the material's stability and rate capability.

### 3.3 Dunn's model calculation for evaluation of charge storage mechanism

Since variations in scan rates or current density affect the underlying process, Dunn's model can be used to effectively evaluate the charge storage mechanism at various scan rates. The power law was applied to the CV plots at each scan rate, which ranged from 5 to 50 mV s^−1^, in order to examine the current response.^40^

5 *I*_p_ = *aʋ*^b^

Or

6 Log *I*_p_ = Log *a* + *b* Log (*ʋ*)

Or7
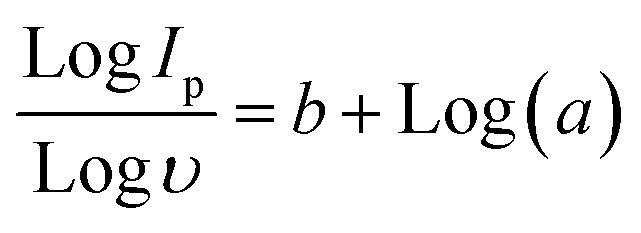


**Fig. 6:**
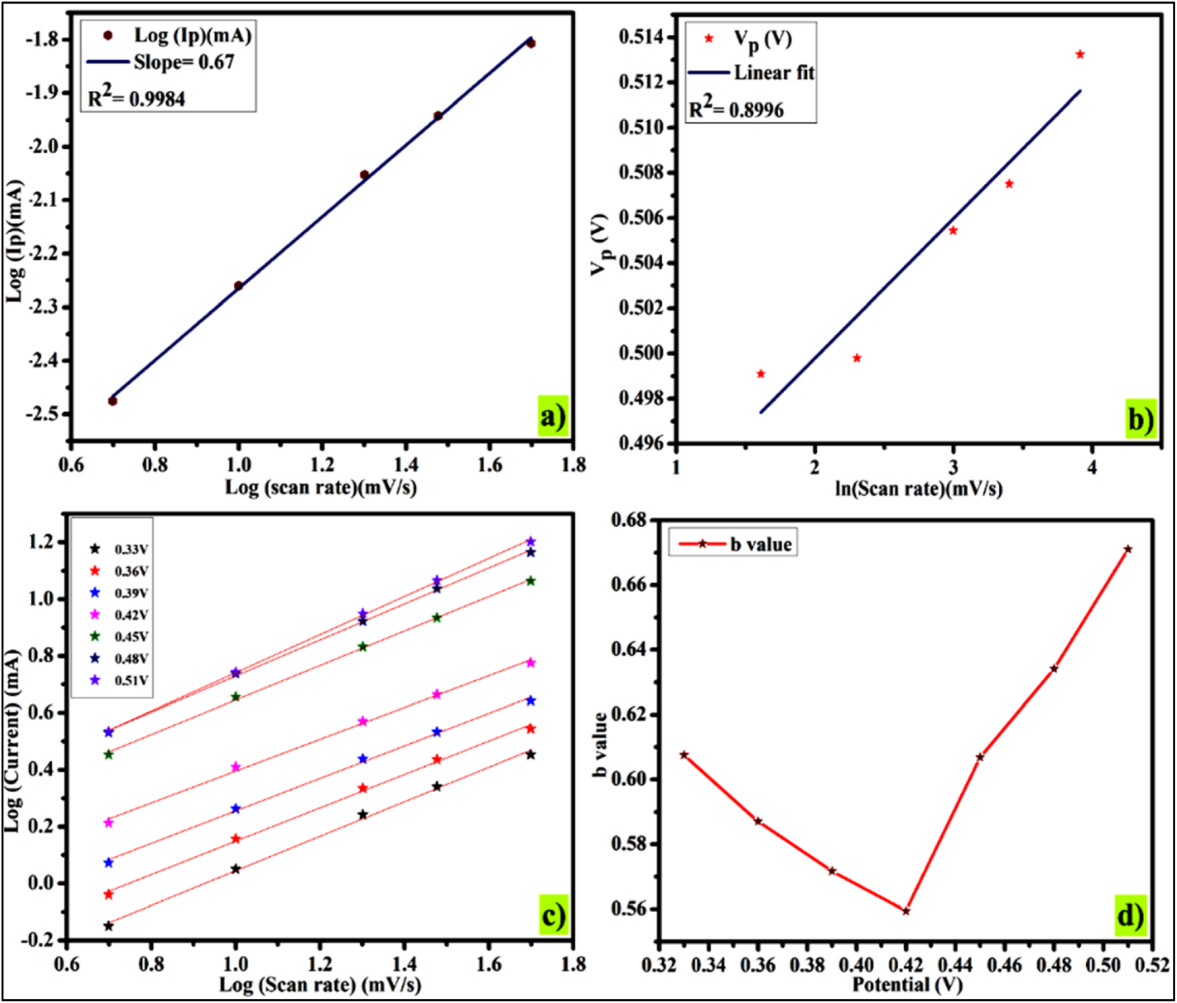
(a) log *ʋ versus* log *I*_p_, (b) ln (*ʋ*) *versus V*_p_, (c) log (scan rate) *versus* log (oxidation peaks current) and (d) *b* value at various potential.

A linear regression analysis of the logarithmic relationship between current (*I*_p_) and scan rate (*ʋ*) reveals insight into the charge storage mechanism. The slope of logarithmic plot 
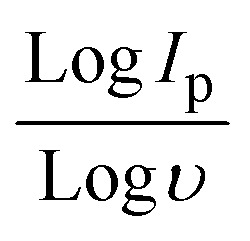
 yields the *b* value, while the *y*-intercept relates to the log a. A *b* value of 0.5 suggests diffusion-controlled behavior, whereas a value of 1 indicates capacitive or non-diffusion-limited kinetics. The obtained *b* value of 0.67 from linear regression analysis indicates battery-grade characteristics, consistent with previous conclusions and findings.^41^ The *b* value indicates the pseudocapacitive phenomenon arises due to the mixed capacitive-type charge storage phenomenon and diffusive-type charge storage phenomenon. The sharp, symmetric redox peaks indicated the transfer of electrons *via* the oxidation and reduction process. The small shift in redox peaks indicated the polarization effects, and the enhanced area under the curve provides the mixed diffusive/capacitive phenomenon Fig. 6(a). Furthermore, a linear correlation between peak potential (*V*_p_) and ln (*ʋ*) suggests enhanced charge and ion transfer *via* diffusion, supporting the aforementioned results Fig. 6(b). The graph displaying log current (*I*) and log scan rate (*ʋ*) to determine *b* values as depicted in Fig. 6(c). At the lowest potential (0.33 V), the *b* value is 0.61, and it decreases as the potential increases. Fig. 6(d) revealed that the *b* value is smallest at 0.42 V, which rises with the rise in values of scan rate. The *b* value suggests that the current originates from a capacitive mechanism, which generally shifts to a diffusion-dominated process as the scan rate increases.^42^ The maximum achievable current stems from the diffusion-controlled process, particularly the ion-intercalation process that is occurring between the layers of electrode material.^43^

The following relation is used to quantitatively evaluate the diffusive and capacitive current;^44^

8 *I*(*v*) = *I*_cap_ + *I*_diff_

9 *I*(*V*) = *K*_1_*ʋ* + *K*_2_*ʋ*_2_^1^

Or10
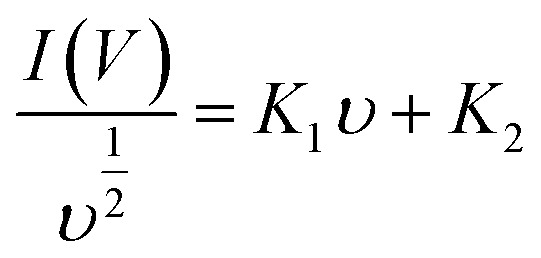


**Fig. 7:**
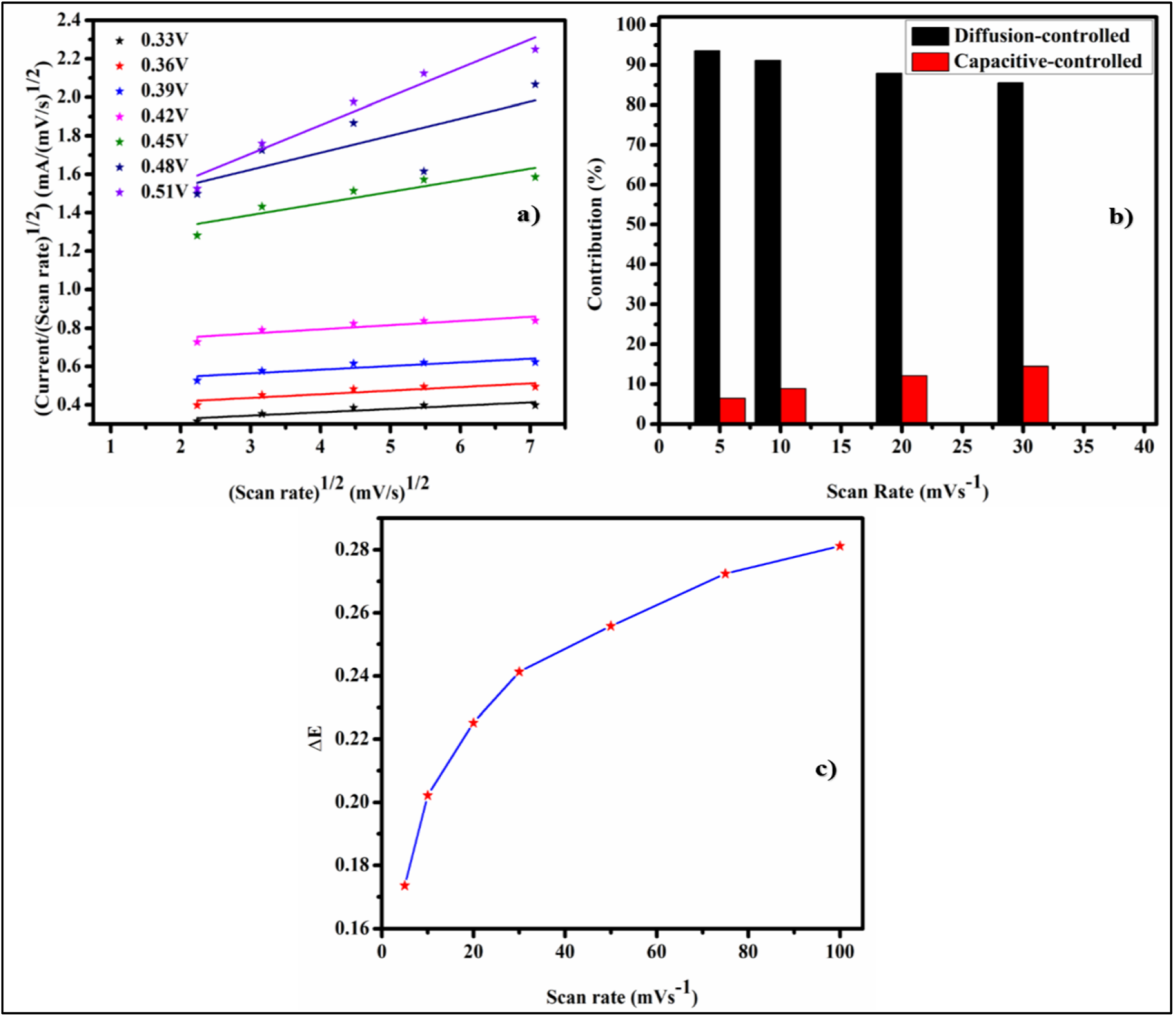
(a) The plot of *ʋ*^1/2^*vs. I*/*ʋ*^1/2^ for evaluation of *K*_1_ and *K*_2_ values, (b) plot of current contribution *versus* scan rate (c) peak potential separation *versus* scan rate.

As illustrated in Fig. 7(a), the *K*_1_ (slope) and *K*_2_ (*y*-intercept) values were determined from the linear fit by plotting *I*(*v*)/*ʋ*^1/2^ against *ʋ*^1/2^. As shown in Fig. 7(b), the capacitive current increases as the scan rate increases because there is less time for diffusion among the electrode material layers. Consequently, non-diffusion-limited behavior predominantly governs the charge storage mechanism at elevated scan rates. Moreover, the gap of redox peak potentials increases with rising scan rates, attributable to the dielectric polarization effect as depicted in Fig. 7(c).

### 3.4 Galvanostatic charging/discharging measurements

**Fig. 8:**
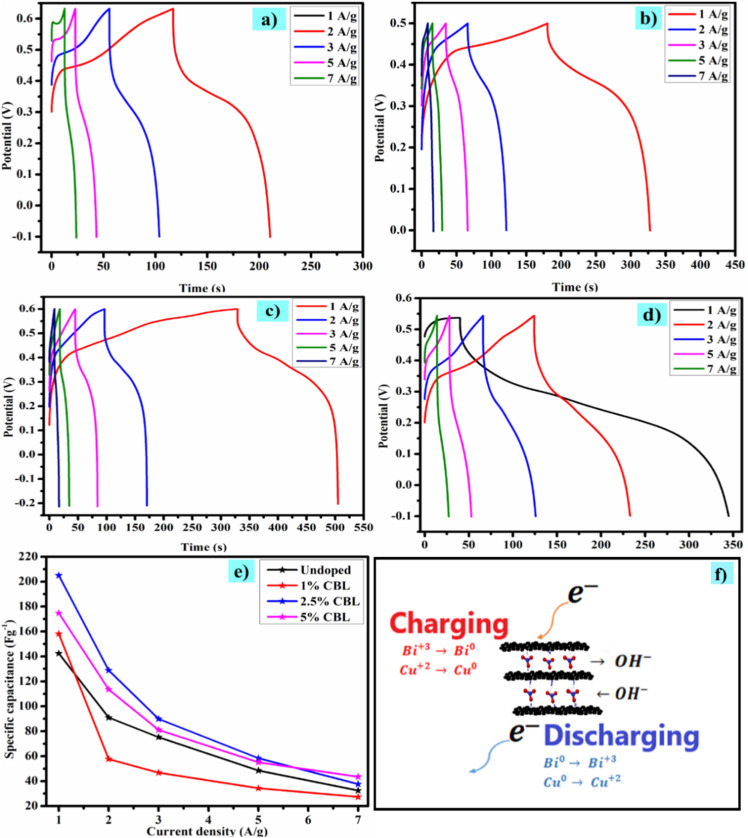
GCD curves (a) undoped material, (b) 1% CBL, (c) 2.5% CBL, (d) 5% CBL, (e) *C*_p_ of synthesized electrode materials at various current densities (f) supposed redox reaction mechanism of 2.5% CBL.

The galvanostatic charging/discharging analysis was carried out at 1, 2, 3, 5, and 7 A g^−1^ current densities to further evaluate the charge-storing phenomenon. The shape of the GCD curve of 2.5% CBL suggests the battery-type nature, which further supports the results obtained by applying Dunn's model on CV data as discussed above (Fig. 8 showcasing the GCD curves of synthesized materials).^45^

Following relation was used to calculate the specific capacitance for each electrode material;11
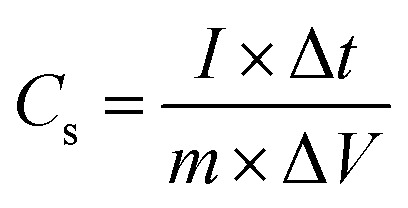
Here *I* denotes the current applied in mA, Δ*t* indicates the discharging time, *m* represents the mass of active material loaded on NF (nickel foam), and the operating potential window is represented by Δ*V*, energy density and power density were measured by utilizing eqn (12) and (13);^46^12
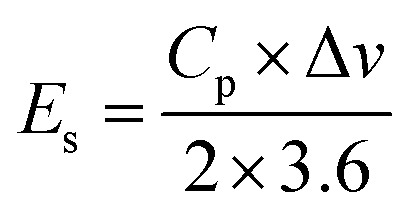
13
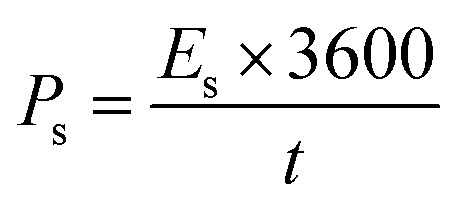
In this relationship, *E*_s_ (Wh kg^−1^) denotes the energy density (W kg^−1^), *P*_s_ represents the power density, *C*_p_ represents the specific capacitance (F g^−1^) and the operational potential window is represented by Δ*V*.

Improved (highest of all the materials examined) *C*_p_ (205 F g^−1^) for 2.5% CBL is obvious from Fig. 8(e), which might be due to better incorporation of copper in the bismuth interface and layered structure of the material.^47^ Further, it can be clearly observed that with increasing current density, specific capacitance decreased due to less time available for intercalation of ions within the host layers. Schematics of possible redox reactions that occur during the overall charge/discharge process is shown in Fig. 8(f).

### 3.5 Electrical impedance spectroscopy measurements

**Fig. 9:**
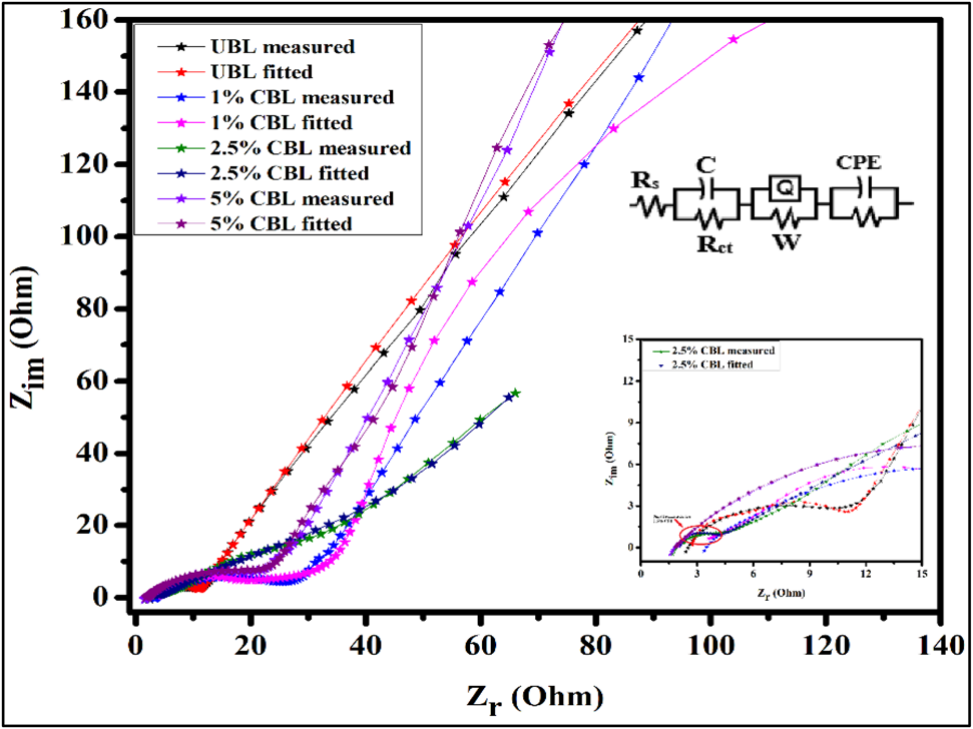
Comparison of Nyquist plots of synthesized materials, inset: electrical circuit model from ZSimpWin software, and a higher frequency region of Nyquist plots.

To assess the resistive and charge transfer properties of the electrode material, electrochemical impedance spectroscopy (EIS) was conducted, and the resulting Nyquist plots for undoped and copper-doped bismuth layered double hydroxide (with varying% ratios) are presented in Fig. (9). A frequency range of 10^−2^ to 10^2^ kHz was employed to investigate the impedance and conductive behavior. The Nyquist plot illustrates the relationship between *Z*′-real impedance and –*Z*″ (imaginary impedance), with *R*_s_ representing the solution resistance and series resistance, *R*_ct_ denoting charge transfer resistance, and CPE signifying the electrode materials' internal resistance. In the inset of Fig. (9) a small semicircle is observed for 2.5% CBL, showing the conductive nature of the material. In the case of an undoped material, a large circumference (*R*_ct_) is observed, which might be due to the irregular and non-uniform surface of the material. The lowest value of charge transfer resistance (*R*_ct_) for 2.5% CBL suggested that the rate of ion transportation within the material became faster, proving the material highly conductive. The value of capacitance (*C*_p_) is increased in the case of 2.5% CBL due to the presence of a higher number of active sites. The lowest constant phase element (CPE) value of 2.5% CBL suggested its battery-type supercapacitor electrochemical nature, supporting the results obtained from CV and GCD analysis as discussed above.

Intercalating host ions offered additional conducting pathways for electron transport, and the electrolyte's ions (OH^−^) interacted well with both ions and atoms within the tiny pores of the prepared electrode. Diffusion of ions is linked to the linear portion of the low-frequency area, which is known as the Warburg diffusion impedance. Here we have the solution resistance (*R*_s_), capacitor (*C*), charge transfer resistance (*R*_ct_), Warburg diffusion resistance (*W*), and constant phase element (CPE) shown in that order.

### 3.6 Electrochemical evaluation of the fabricated supercapacitor

**Fig. 10:**
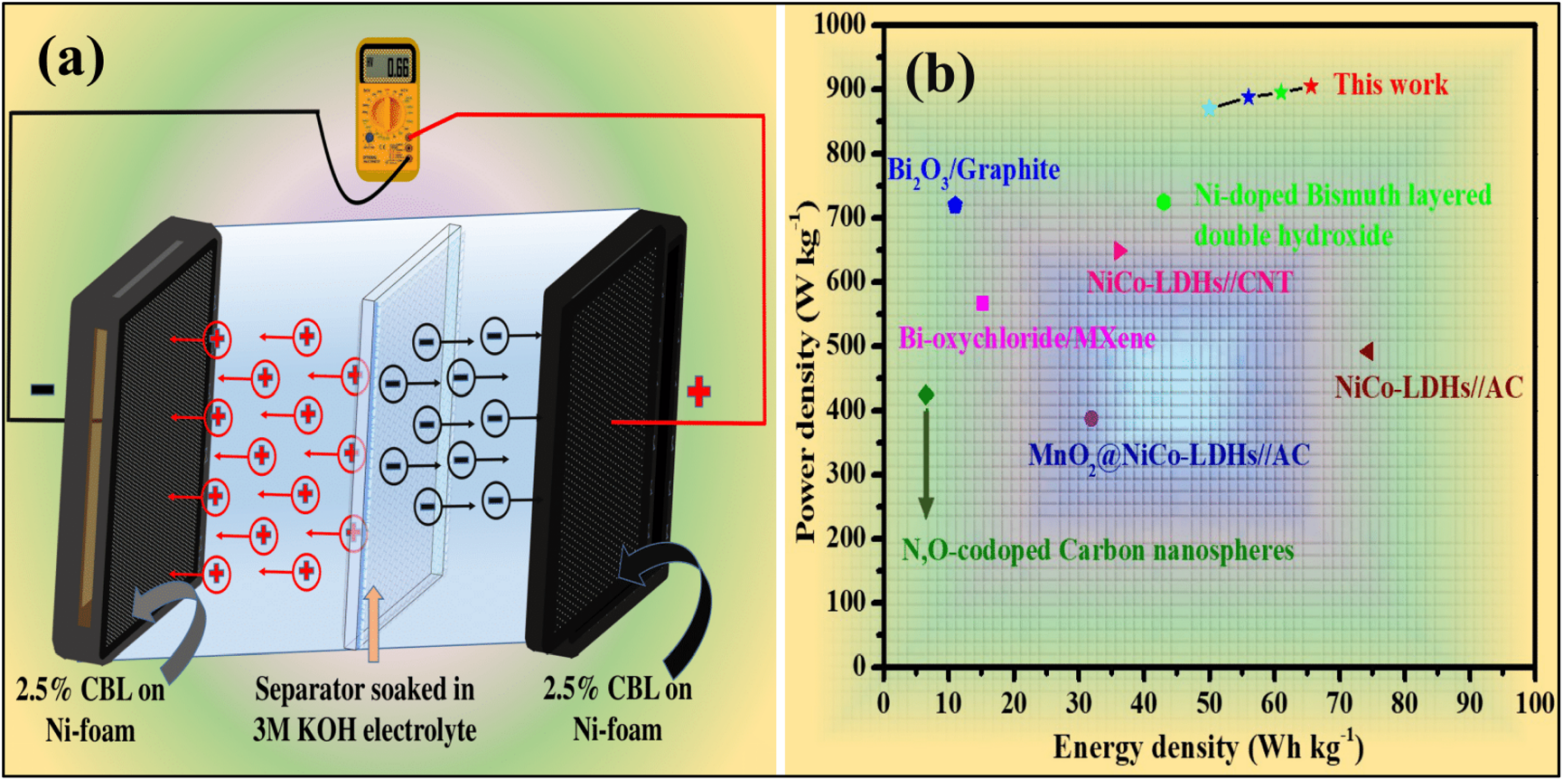
(a) Illustration of a fabricated symmetric pseudocapacitor device, (b) Ragone plot (energy density *versus* Power density).

A symmetric pseudocapacitor device was assembled using 2.5% Cu-doped bismuth layered double hydroxide as both the cathode and anode, with a Whatman filter paper No. 01 impregnated in 3 M KOH serving as the separator (the assembly is shown in Fig. 10(a). The electrochemical performance of the device was evaluated using a two-electrode configuration, where one electrode was connected to the both reference and counter electrode leads, and the other electrode was connected to the working lead.

**Fig. 11:**
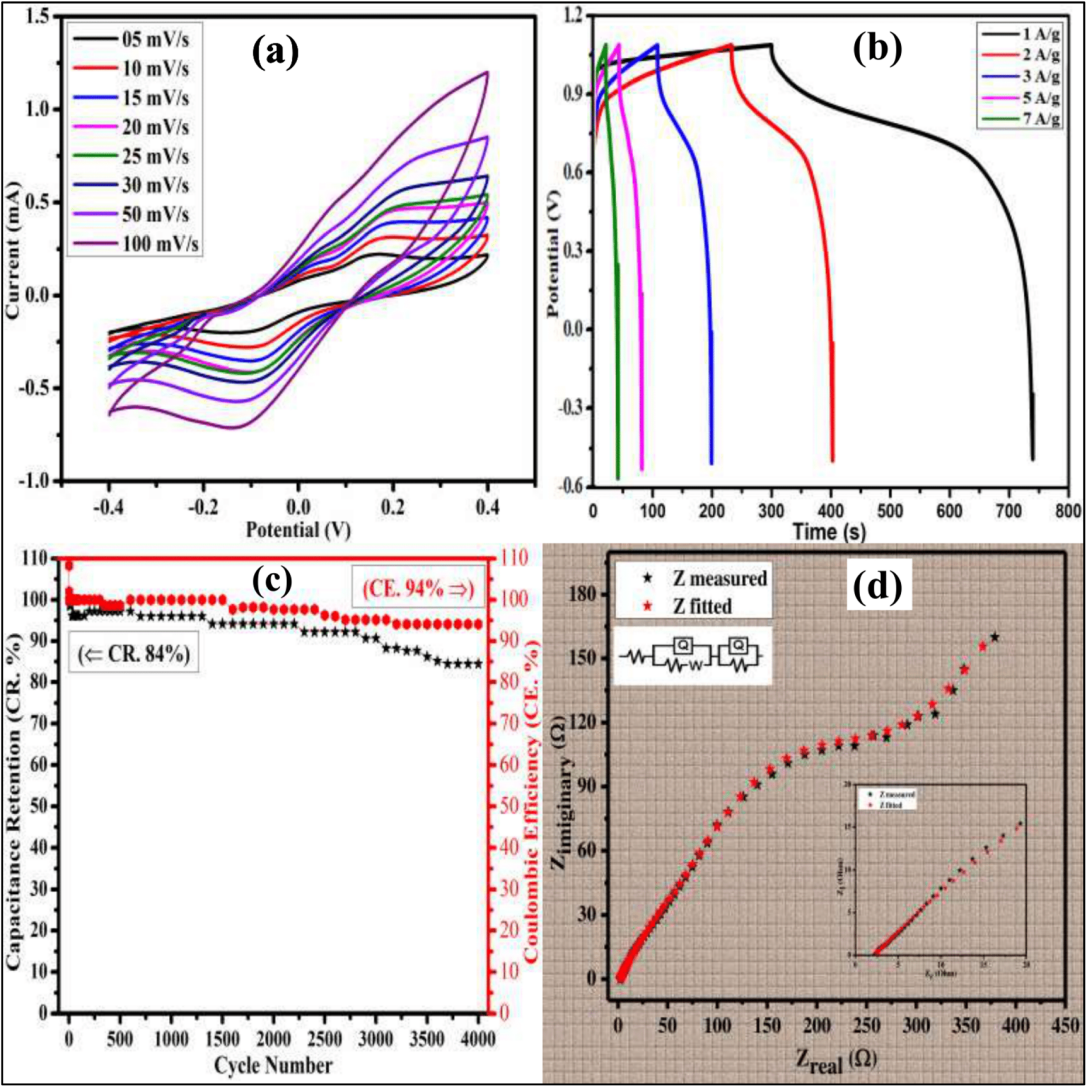
(a) CV curves of device at various scan rates (5, 10, 15, 20, 25, 30, 50 and 100 mV s^−1^) within potential window range of −0.4 V to 0.4 V, (b) galvanostatic charge/discharge curves obtained at current densities 1, 2, 3, 5 and 7 A g^−1^, (c) capacitance retention and coulombic efficiency performance of device at 4000 cycles and (d) Nyquist plot obtained from EIS data (inset: zoomed area at higher frequency region).

The electrochemical properties of the symmetric pseudocapacitor device were comprehensively evaluated using cyclic voltammetry (CV) at scan rates ranging from 5 to 100 mV s^−1^, as displayed in Fig. 11(a). To optimize device performance and mitigate water electrolysis, the potential window was strategically set between −0.4 and 0.4 V. The CV curves exhibited distinct faradaic redox peaks, characteristic of pseudocapacitive behavior, which was further corroborated by Dunn's model calculations. To gain deeper insights into the device's electrochemical behavior, galvanostatic charge/discharge (GCD) analysis was conducted. The GCD curves of the fabricated device displayed non-linear profiles, confirming the pseudocapacitive nature of the material Fig. 11(b). The specific capacitance of the device was calculated from the GCD curves using eqn (11). The device demonstrated impressive specific capacitances of 253.9, 194.3, 156.8, 89.8, and 88.6 F g^−1^ at current densities of 1, 2, 3, 5, and 7 A g^−1^, respectively. Furthermore, the device's long-term stability was assessed through a cyclic life test, comprising 4000 cycles at a current density of 10 A g^−1^. Remarkably, the device retained 84% of its initial capacitance and exhibited a coulombic efficiency of 94% after 4000 cycles as displayed in Fig. 11(c), underscoring its excellent electrochemical stability and potential for practical applications. Fig. 11(d) shows an EIS-fitted Nyquist plot of the constructed device between real impedance (*Z*′) and imaginary impedance (–*Z*″). To gain a deeper understanding of the device's electrochemical behavior, electrochemical impedance spectroscopy (EIS) data were analyzed using ZSmpWin software. The circuit fitting process enabled the extraction of various circuit element values, providing valuable insights into the device's internal resistance and charge transfer kinetics. The Nyquist plot displayed in the inset of Fig. 11(d) revealed a small semicircle, indicative of the charge transfer resistance (*R*_ct_), while the intercept on the real axis represented the equivalent series resistance (ESR). Additionally, the plot exhibited straight lines at a 45° angle, suggesting Warburg diffusion between the electrode layers.^48,49^

The Ragone plot (Fig. 10(b)) illustrates the relationship between energy density and power density, offering a comprehensive evaluation of the device's performance. A comparative analysis with previously reported devices fabricated from similar materials (Fig. 10(b)) demonstrates that the fabricated device achieved superior specific energy while maintaining its specific power. This enhanced performance underscores the device's potential for practical applications in energy storage.

### 3.7 Electrochemical measurements as a function of temperature

To analyze the thermal stability and performance of electrode material at higher temperatures, electrochemical measurements were carried out under different temperature conditions (*i.e.*, 35 °C, 45 °C, and 55 °C). The behavior of battery-type supercapacitor materials obviously varies with increasing or decreasing temperature.^46^ At higher temperatures, the movement of the electrolyte's ions becomes faster, and the ion diffusion process occurs rapidly. Furthermore, when the temperature is excessively increased, the capacitor's performance is lowered due to the low density of electrolyte, excess of electrolyte's ions causing a hindrance in net charge transfer, thereby reducing the performance. Higher temperature mostly results in the superior electrochemical characteristics *i.e.* enhanced capacitance and lower impedance. It might be due to higher ionic conductivity, reduced resistance, and better ion adsorption/desorption rate at higher temperatures.

**Fig. 12:**
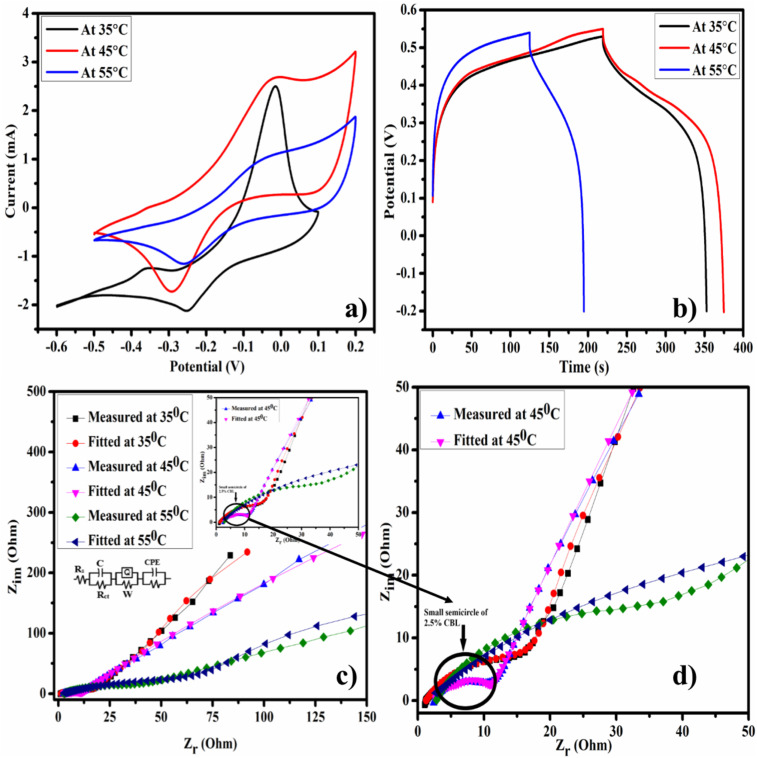
Effect of temperature on electrochemical measurements: (a) CV curves of 2.5% CBL at different temperatures, (b) GCD curves of 2.5% CBL at 35 °C, 45 °C, and 55 °C, (c) Nyquist plots of 2.5% CBL at different temperatures, and (d) higher frequency region showing a very small semicircle at 45 °C.

Fig. 12(a) explores the CV responses of 2.5% CBL at different temperature conditions (35 °C, 45 °C, and 55 °C) at a scan rate of 5 mV s^−1^. As the temperature increased from room temperature to 35 °C, the movement of ions became faster and resulted in better current response. As the temperature further increased to 45 °C, the movement of ions became regular and resulted in enhanced specific capacitance. But further increase in temperature to 55 °C resulted in lower current response and decreased capacitance, which might be due to the hindrance created by the excess of electrolyte ions at elevated temperature. Further investigation of the temperature influence was explored by GCD analysis of 2.5% CBL at different temperatures. Fig. 12(b) depicts that upon increasing the temperature charging–discharging response of the electrode material became very prominent. It might be the result of higher ion diffusion across the electrode.^50^ Furthermore, it was observed that 2.5% CBL gave highest specific capacitance (201 F g^−1^) at 45 °C and lowest one (147 F g^−1^) at 55 °C. Combination of superior energy density and power density was observed at 45 °C.

Temperature more significantly effects the resistance and conductivity of the electrode material is shown in Fig. 12. It was observed that value of charge transfer resistance decreased pronouncedly upon increasing the operating temperature. Zoom area of fitted Nyquist plots at three different conditions of temperature depict the smallest semicircle of 2.5% CBL at 45 °C Fig. 12(d). Moreover, it was observed that quality of fitting equivalent circuits obviously varies with temperature as the circuit which best fit for Nyquist plot at 45 °C doesn't fit well for Nyquist plot at 55 °C.^50,51^

## 4. Conclusion

This study reports a sustainable and economical method for synthesizing 2D heterostructure copper-doped bismuth layered double hydroxide (2.5% CBL, approximately 1.9 µm in size), verified through scanning electron microscope (SEM) and UV-vis spectroscopy. Due to better intercalation and deintercalation of ions, 2.5% CBL worked at its best performance (205 F g^−1^) within the optimized potential window range and depicted greater energy density and power density than its related materials, which are previously reported. The enhanced conductivity of 2.5% CBL can be attributed to the improved ion mobility within its multilayered structure. Temperature treatment of electrochemical analysis illustrated that supercapacitor performance can be enhanced by increasing the temperature to a limited extent due to enhanced ionic movement. An extensive increase in temperature lowered the performance due to the hindrance caused by the rush movement of ions. It can be concluded that due to the pseudocapacitive asymmetric nature of CBL, as confirmed by applying Dunn's Model, 2.5% CBL can be a better choice as an electrode material for symmetric pseudo-capacitors.

## Author contributions

Muhammad Farooq Rasheed: writing – original draft preparation, Yasir Altaf: supervision, Muhammad Ramzan Khawar: writing – original draft preparation, Sajad Hussain: supervision, Najam Ul Hassan: software, validation, Razan A. Alshgari: visualization, investigation, Mohamed Ouladsmane: funding acquisition, Dongwhi Choi: supervision, Awais ahmad: writing – reviewing and editing.

## Conflicts of interest

The authors declare that they have no known competing financial interests or personal relationships that could have appeared to influence the work reported in this paper.

## Supplementary Material

RA-016-D5RA08811A-s001

## Data Availability

All datasets generated and/or analyzed during the current study are available within the article and supplementary information (SI). Supplementary information: Table S1: measurement of resistance from EIS data for three electrodes. Table S2: comparison of current research with similar materials. Table S3: measurement of resistance from EIS data for two electrodes at different temperatures. See DOI: https://doi.org/10.1039/d5ra08811a.
