# Supplementary material for: Effect of dopant concentration and ambient temperature on the pseudo-capacitance behavior of novel copper doped bismuth layered double hydroxide
Source: RSC Adv. 2026 Jan 28;16(7):6032–47. doi: 10.1039/d5ra08811a (PMC12849026; doi:10.1039/d5ra08811a)
Supplement: RA-016-D5RA08811A-s001 [file RA-016-D5RA08811A-s001.pdf]

Supplementary Information (SI) for RSC Advances.
This journal is © The Royal Society of Chemistry 2026

Supporting information

Effect of Dopant Concentration and Ambient Temperature on the Pseudo-capacitance Behavior of Novel Copper Doped Bismuth Layered Double Hydroxide

Muhammad Farooq Rasheed[1], Yasir Altaf[1], Muhammad Ramzan Khawar[3], Sajad Hussain[2], Najam Ul Hassan[2], Razan A. Alshgari[4], Mohamed Ouladsmane[4], Dongwhi Choi[3*]Awais ahmad[4*]*

[1]*Department of Chemistry, Division of Science and Technology, University of Education, Lahore 54000, Pakistan.*

[2]*Department of Physics, Division of Science and Technology, University of Education, Lahore 54000, Pakistan.*

[3]*Department of Mechanical Engineering (Integrated Engineering Program), Kyung Hee University, 1732 Deogyeong-Daero, Yongin, Gyeonggi, 17104, South Korea*

[4]*Department of Chemistry, P. O. Box 2455, College of Science, King Saud University, Riyadh 11451, Saudi Arabia*

[5]*Department of Chemistry, The University of Lahore, Lahore, Pakistan*

Corresponding author:

Sajad Hussain* (sajad.hussain@ue.edu.pk) dongwhi choi* (dongwhi.choi@khu.ac.kr) Awais ahmad* (awaisahmed@gcuf.edu.pk)

Table S1: Measurement of resistance from EIS data for three electrodes

Electrode	Solution resistance (Ohm)	Charge transfer resistance (Ohm)	Series resistance (Ohm)
UBL	0.29	10.5	10.84
1 % CBL	0.31	4.0	4.31
2.5 % CBL	0.29	3.92	4.21
5 % CBL	0.21	22.12	22.33

Table S2: Comparison of current research with similar materials

Structure	Type	Electrolyte	Energy ($Wh\ kg^{-1}$)	Power ($W\ kg^{-1}$)	References
N, O codoped carbon nanosphere	Symmetric	KOH	6.5	425	[1]
Bi-Oxychloride/Mxene	Symmetric	KOH	15.2	567.4	[2]
Bi_2O_3/Graphite	Asymmetric	KOH	11	720	[3]

Ni-Bismuth LDH	Symmetric	KOH	43	725	[4]
NiCo-LDHs//AC	Asymmetric	KOH	74.37	492	[5]
NiCo-LDHs//CNT	Asymmetric	KOH	36.1	649	[6]
MnO_2@CoNi-LDHs//AC	Asymmetric	KOH	40.9	400	[7]
Cu-Bismuth LDH	Symmetric	KOH	65.5	905	This work

Table S3: Measurement of resistance from EIS data for two electrodes at different temperatures

**Temperature**	**Solution resistance (Ohm)**	**Charge transfer resistance (Ohm)**	**Series resistance (Ohm)**
35	2.25	19.01	21.06
45	2.22	11.12	13.34
55	2.29	70	70.29

1. Hu, M., et al., *Emerging 2D MXenes for supercapacitors: status, challenges and prospects.* Chemical Society Reviews, 2020. **49**(18): p. 6666-6693.

2. Gogotsi, Y. and Q. Huang, *MXenes: two-dimensional building blocks for future materials and devices*. 2021, ACS Publications. p. 5775-5780.

3. Chodankar, N.R., et al., *True meaning of pseudocapacitors and their performance metrics: asymmetric versus hybrid supercapacitors.* Small, 2020. **16**(37): p. 2002806.

4. Attia, S.Y., et al., *A two-dimensional nickel-doped bismuth-layered double hydroxide structure as a bifunctional efficient electrode material for symmetric supercapacitors.* Sustainable Materials and Technologies, 2023. **36**: p. e00595.

5. Liu, H., et al., *Controlled microstructure in two dimensional Ni-Co LDH nanosheets-crosslinked network for high performance supercapacitors.* Advanced Powder Technology, 2019. **30**(6): p. 1239-1246.

6. Ramachandran, R., et al., *Construction of NiCo-layered double hydroxide microspheres from Ni-MOFs for high-performance asymmetric supercapacitors.* ACS Applied Energy Materials, 2020. **3**(7): p. 6633-6643.

7. Luo, H., et al., *Hierarchical design of hollow Co-Ni LDH nanocages strung by MnO2 nanowire with enhanced pseudocapacitive properties.* Energy Storage Materials, 2019. **19**: p. 370-378.
